# Single Crystal Sub‐Nanometer Sized Cu_6_(SR)_6_ Clusters: Structure, Photophysical Properties, and Electrochemical Sensing

**DOI:** 10.1002/advs.201600126

**Published:** 2016-07-14

**Authors:** Xiaohui Gao, Shuijian He, Chunmei Zhang, Cheng Du, Xi Chen, Wei Xing, Shengli Chen, Andre Clayborne, Wei Chen

**Affiliations:** ^1^ State Key Laboratory of Electroanalytical ChemistryInstitution Changchun institute of Applied ChemistryChinese Academy of Sciences5625 Renmin StreetChangchun130022JilinChina; ^2^ University of Chinese Academy of Sciences100039BeijingChina; ^3^ Department of ChemistryNanoscience CenterUniversity of JyväskyläJyväskyläFI‐40014Finland; ^4^ College of Chemistry and Molecular SciencesWuhan UniversityWuhan430072China

**Keywords:** copper, electrochemical sensor, nanocluster, optical absorption, single crystal

## Abstract

Organic ligand‐protected metal nanoclusters have attracted extensively attention owing to their atomically precise composition, determined atom‐packing structure and the fascinating properties and promising applications. To date, most research has been focused on thiol‐stabilized gold and silver nanoclusters and their single crystal structures. Here the single crystal copper nanocluster species (Cu_6_(SC_7_H_4_NO)_6_) determined by X‐ray crystallography and mass spectrometry is presented. The hexanuclear copper core is a distorted octahedron surrounded by six mercaptobenzoxazole ligands as protecting units through a simple bridging bonding motif. Density functional theory (DFT) calculations provide insight into the electronic structure and show the cluster can be viewed as an open‐shell nanocluster. The UV–vis spectra are analyzed using time‐dependent DFT and illustrates high‐intensity transitions involving primarily ligand states. Furthermore, the as‐synthesized copper clusters can serve as promising nonenzymatic sensing materials for high sensitive and selective detection of H_2_O_2_.

## 1 Introduction

Thiolate‐stabilized metal nanoclusters (size less than 2 nm) typically consist of metallic cores with tens to several hundreds of metal atoms, which are surrounded by thiolate ligands ranging in size and composition. This special class of nanocluster has received enormous attention because of their unique chemical and physical properties with potential applications in catalysis, chemical sensors, nanodevices, etc.^1^ Due to the atomically precise composition and determined atom packing structure, thiolate‐stabilized metal nanoclusters have become an ideal system for studying the structure and size‐dependent properties of metal particles. Similarly, these systems have shown some very interesting quantum size effect and core size‐dependent optical and electronic properties.^2^ Among those studied, most research has been focused on thiol‐stabilized gold and silver nanoclusters (sometimes referred to as monolayer protected nanoclusters). Up to now, different research groups have successfully synthesized a variety of thiol‐stabilized Au (and Ag) nanoclusters that range in size and composition with various types of protecting ligands (for reviews of thiol‐stabilized nanoclusters please see ref. 3). However, due to the lower stability and easier oxidation compared to Au and Ag, much less research has been carried out on copper nanoclusters.^4^

On the other hand, determination of crystal structures of metal nanoclusters is of crucial importance to understand the unique properties and exploit the related applications. Growth of high‐quality single crystals of metal nanoclusters is the first step to examine their crystal structures with well‐defined metal atom packing and metal–ligand bonding. Over the past decades, as an important class of noble metal nanomaterials, a number of specific single crystals of gold nanoclusters have been successfully synthesized and the crystal structures have been well characterized by various analytic techniques, including mass spectrometry, optical spectrometry, and X‐ray crystallography.[[qv: 2c,5]] In parallel with gold nanoclusters, several silver and alloy nanoclusters have also been identified and a few of them have been structurally analyzed, such as Ag_44_, Au_32_Ag_12_, etc.[[qv: 1c,6]] Compared to gold and silver, the determination of precise crystal structure of copper nanoclusters is still lacking and almost all the current reports limit in the rough synthesis and ambiguous characterization due to its susceptibility to oxidation upon exposure to air and the difficulty of single crystal growth.[[qv: 4a,b,7]] In recent years, copper nanoclusters, including complexes, are experiencing a resurgence of interest for researchers. This has been due in part to the drive to determine and understand the growth of the nanocluster's crystal structure, along with understanding the photophysical properties for materials, as well as the involvement of various cluster compounds at the active site of biomolecules. Previous experimental studies on copper nanoclusters surrounded by ligands have shown interesting structural, electrochemical, and photophysical properties. However, the precise geometry of the ligand stabilized systems could only be obtained through theoretical analysis but not confirmed through single crystal characterization.

Although some copper complexes have been reported and their structures have been characterized, the optical properties and applications of copper nanoclusters are still scarce.^8^ Herein, we report the successful synthesis of thiol‐stabilized Cu_6_ nanoclusters and their single crystals (Cu_6_(C_7_H_4_NOS)_6_) with mercaptobenzoxazole as protecting ligands and the determination of its single crystal structure. In a synergistic effort with experiment and theory, we also investigated the optical properties of (Cu_6_(C_7_H_4_NOS)_6_)^−^ and explain the origin of excitation in the optical spectra. Moreover, we illustrate that the hexanuclear copper nanoclusters could be used as a class of efficient electrochemical sensing materials for the detection of H_2_O_2_.

## 2 Results and Discussion

### 2.1 Synthesis and Characterization of Cu Nanoclusters

**Figure 1:**
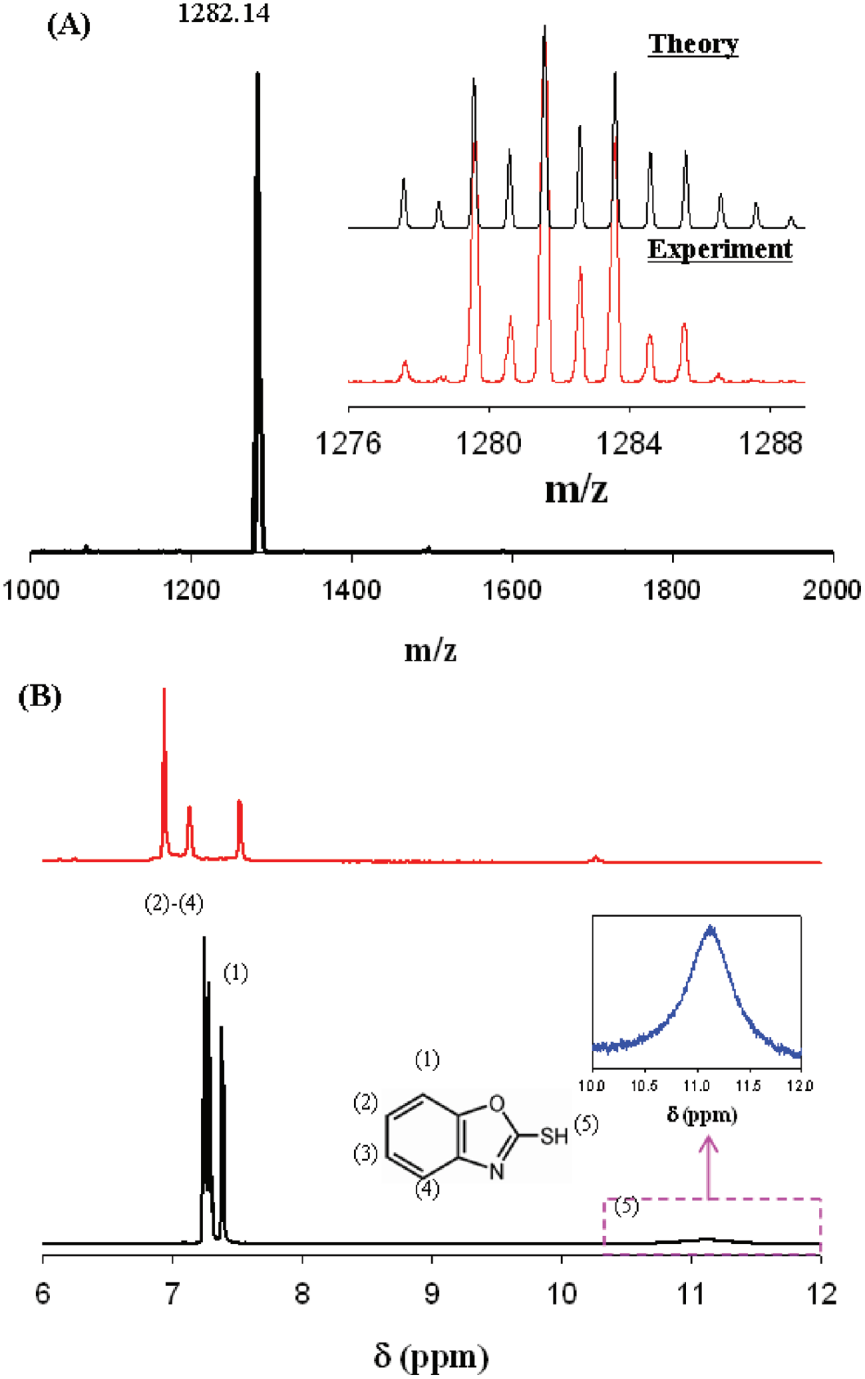
A) ESI‐MS of Cu_6_(SR)_6_ clusters. Inset compares the experimental and simulated isotopic MS patterns. B) ^1^H NMR spectra of Cu_6_(SR)_6_ (red curve) and 2‐mercaptobenzoxazole monomer (black curve). The left‐bottom inset shows the molecular structure of 2‐mercaptobenzoxazole (MBO) and the right‐bottom inset shows the enlarged view of the pink rectangle part.

The composition and charge state of the as‐synthesized copper clusters were first characterized by electrospray ionization mass spectrometry (ESI‐MS). As shown in **Figure** **1**A, a single pronounced peak of parent cluster ions appears at *m/z* = 1282.14 in a negative ion mode, indicating the high purity and singly negative charge of the synthesized clusters. Based on the mass peak, a chemical formula of Cu_6_ (C_7_H_4_ONS)_6_^−^ could be assigned to the formed Cu sub‐nanometer clusters. Meanwhile, the experimental isotopic pattern of the clusters overlaps perfectly with the simulated one, as shown in Figure 1A inset. Figure 1B shows the ^1^H NMR spectra of the produced Cu_6_(C_7_H_4_ONS)_6_ clusters and free 2‐mercaptobenzoxazole ligands. Apparently, the proton signal from the**‐**SH around 11.4 ppm disappears in the NMR spectrum of Cu clusters because of the formation of S—Cu bond and the cleavage of S—H bond. In parallel, with mercaptobenzoxazole ligand bound onto the cluster surface, the sharp peaks originated from the H atoms on the benzene ring evolve into three segregative, low‐resolution, and a broad hump with the ratio of 1:1:2 under the influence of copper core. Note that in the NMR spectrum of cluster sample, the peaks from 2‐H, 3‐H, and 4‐H move down while the peak from 1‐H shows an up‐shift compared to the NMR of free ligand. These NMR features could be ascribed to the electronic effect from metal core and such observations are consistent with the previous reports.[[qv: 6e,9]] Further insight into the surface state and chemical bonds of the Cu clusters could be acquired from the IR and XPS (X‐ray photoelectron spectroscopy) measurements. In the IR spectrum of copper clusters (Figure S1, Supporting Information), the peak at 2848 cm^−1^ attributed to —S—H stretching vibration is absent while other absorptions from C—C and C—H are present in comparison with the IR spectrum from free ligands, again indicating the formation of Cu—S and the S—H bond breaking. The IR results agree well with the NMR measurements. The XPS survey spectrum (Figure S2A, Supporting Information) shows the presence of Cu, S, N C, and O in the cluster product. The corresponding high resolution S 2p and Cu 2p spectra are displayed in Figure S2B,C of the Supporting Information. From the Cu 2p XPS spectrum, the binding energies of Cu 2p_3/2_ and Cu 2p_1/2_ are at 931.3 and 951.3 eV, respectively, indicating the absence of Cu^2+^. However, due to the indiscernible binding energy of Cu(0) and Cu(I) and the possible charge transfer in Cu‐ligand bonds, the oxidation state of Cu_6_ cluster is speculated to be between 0 and +1.

**Figure 2:**
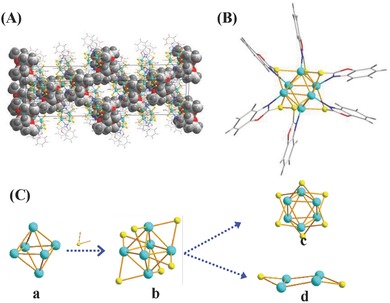
Crystal structure of a 2‐mercaptobenzoxazole‐protected Cu_6_(SR)_6_^−^ nanocluster. A) The structure cell of Cu_6_(SR)_6_^−^ with Na(C_3_H_6_O)_6_^+^ as counterions. The Na(C_3_H_6_O)_6_^+^ ions are displayed in the space‐filling style for clear observation. B) The total structure of a Cu_6_(C_7_H_4_ONS)_6_ cluster. Color legend: green sphere, Cu; yellow sphere, S; blue sphere, N; red sphere, O; light gray sphere, C; deep gray sphere, H. C) The core structure of a Cu_6_ cluster (a); the Cu_6_S_6_ framework (b); the top view of Cu_6_S_6_ framework (c); and chair‐like conformation (d).

**Figure** **2** shows the crystal structure of the obtained Cu_6_ clusters. According to the single‐crystal analysis, the unit cell falls into the hexagonal crystal system and R‐3c space group, as shown in Figure 2A and Figure S3 of the Supporting Information. In the crystal packing structure, the rigid and large acetone‐coordinated sodium ions (Na(C_3_H_6_O)_6_^+^, cuboctahedron) exist as counterions that occupy the space between the clusters, and keep the clusters from free rotation, which results in the successful crystallization. Notably, ICP‐MS measurements from the crystal solution demonstrate the existence of sodium ions. *Zeta* potential measurement (−23.3 mV) also showed the negatively charged state of the clusters. Obviously, each cluster compound is equipoised with single Na(C_3_H_6_O)_6_^+^ and holds a single negative charge. The detailed parameters of unit cell are given in Table S1 of the Supporting Information.

Figure 2B displays the total structure of a single Cu_6_(SR)_6_ cluster. The Cu_6_ metal core (Figure 2C) can be viewed as a slightly distorted octahedron with six copper atoms and the bonds of Cu—Cu constructing the arris of 2.58–2.88 Å (Table S2, Supporting Information). Each copper atom in the core is coordinated with its adjacent four copper atoms. Similar to the previously reported hexanuclear Cu(I) complexes, the thiolates are positioned in a bridging bonding mode (Figure 2C). Though this bonding has been observed for other copper systems, such binding is atypical for other thiolate‐protected nanocluster systems, which exhibit the staple motif, i.e., the well‐known linear oligomeric unit (RS(Au‐SR)_n_) and 3D units (Ag_2_(SR)_5_).[[qv: 6b,10]] In the present Cu_6_ cluster, each sulfur atom forms a bond to two copper atoms with two Cu—S bond lengths of 2.33 and 2.40 Å, resulting in a short distance between two copper atoms. Meanwhile, two S atoms located at the opposite vertices of octahedron and four copper atoms are bonded by a chair‐like conformation (Figure 2C). Further, the nitrogen atoms form an additional bond with the copper atoms, thus enhancing their stability and compatibility. Moreover, if the Cu_6_ core is considered as square bipyramid, the present cluster has a similar structure to Au_7_L_7_^+^ with pentagonal bipyramid shape.^11^ Meanwhile, based on the crystal structure, the prepared Cu cluster, including the metal core and protecting ligands, has a size of 1.68 nm in length and 1.45 nm in width in a plane, as shown in Figure S4 of the Supporting Information. For only the metal core, the size of the cluster is on sub‐nanometer scale (i.e., −0.37 and 0.27 nm in length and width, respectively).

**Figure 3:**
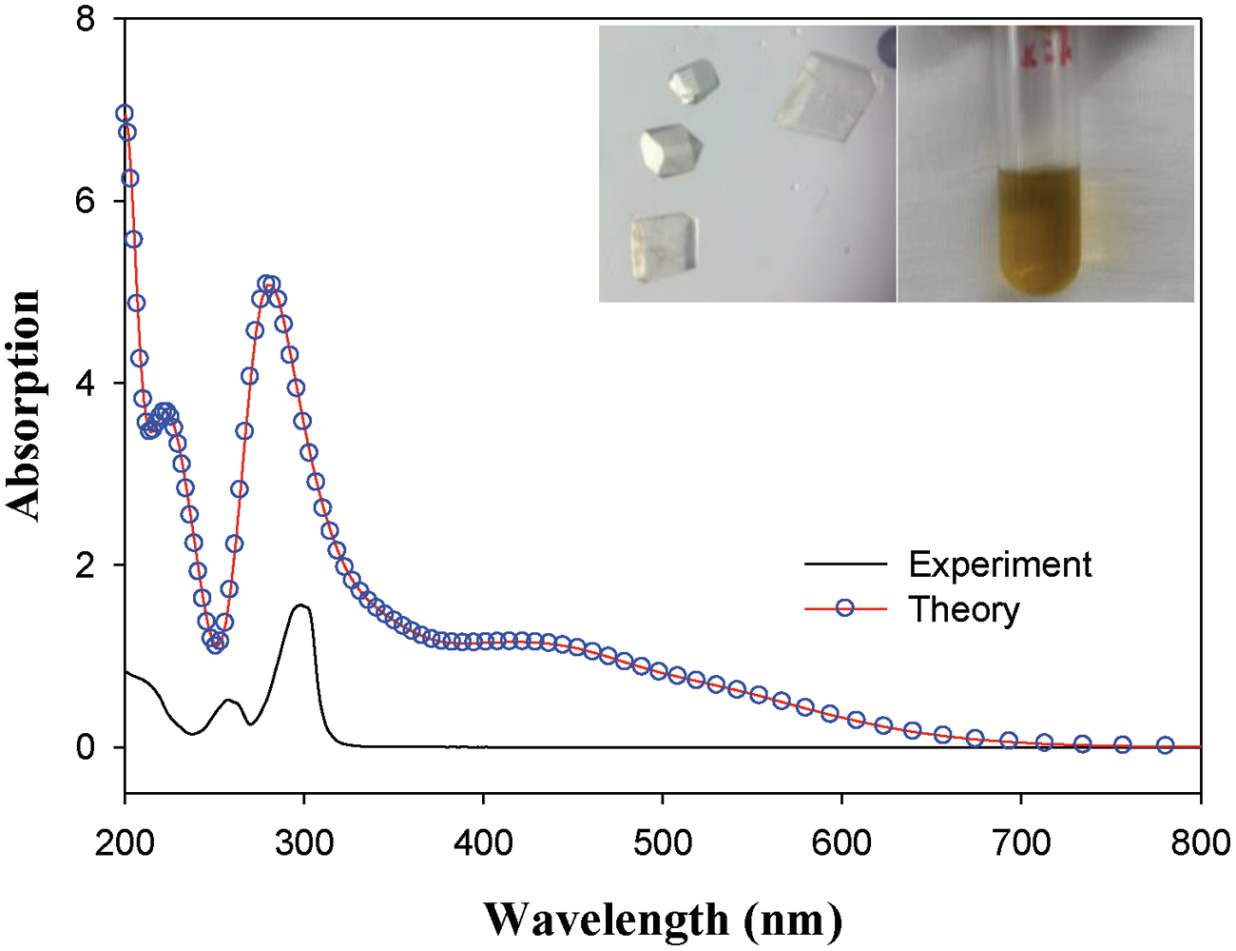
Experimental (black curve) and theoretically predicted (red line + blue circles) UV–vis absorption spectra of the Cu_6_(SR)_6_ nanoclusters. Insets show the photographs of the produced single crystals (left) and dichloromethane solution of Cu_6_ clusters (right).

**Figure** **3** shows the UV–vis absorption spectra of the synthesized Cu_6_(SR)_6_ nanoclusters from experiment in dichloromethane (black curve). Figure 3 insets show the photographs of the produced single crystals (left) and dichloromethane solution of Cu_6_ clusters (right). The experimental spectrum features two absorption peaks at 258 and 299 nm. To obtain further insight into the optical excitations, we evaluated the optical spectra using linear response DFT simulations. Interestingly, the simulated optical spectra of the Cu_6_^−^ nanocluster yields two major peaks at 222 and 284 nm, which taking into account the known error with DFT, is in good agreement with the experimental spectra. However, the simulated spectrum produces a smaller broad absorption at longer wavelengths (400–600 nm). It is well known that for many systems, the crystal structure may differ from those found in solution; therefore, we also investigated the optical spectra for the neutral and −2 states of the Cu_6_ nanocluster (Figure S5, Supporting Information). The simulated spectra of each cluster also agree well with the observed experimental spectra, with a main peak observed at 284 nm. However, similar to the singly negatively charged state, there is a low intensity broad band seen at 450 nm. Equally as important is the role of the solvent on the optical spectra. Our calculations show that the solvent has little to no effect on the optical spectra (Figure S6, Supporting Information). Except for the absorption, the fluorescence properties of the clusters have also been measured and no emission was observed. Note that the emission properties of metal clusters are strongly related to the surface structure and properties, e.g., the surface protecting ligands. In our previous work, the Cu_8_ clusters protected by 2‐mercapto‐5‐n‐propylpyrimidine exhibited strong emissions at 425 and 593 nm, respectively.[[qv: 4b]]

**Figure 4:**
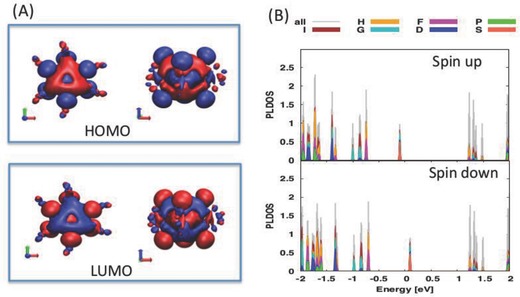
Electronic structure of Cu_6_(SC_7_H_4_NO)_6_. A) The HOMO and LUMO states of Cu_6_(SC_7_H_4_NO)_6_^−1^. B) Projection of the Kohn–Sham electron states to spherical harmonics in the Cu_6_(SC_7_H_4_NO)_6_^−1^ for the spin‐up and spin‐down states.

In order to understand the origin of the optical excitations, we investigated the electronic structure of the Cu_6_ cluster with *q* = −1 and −2. As mentioned previously, the geometry of the crystallized Cu_6_(SR)_6_^−^ cluster is a slightly distorted octahedron, with ligands bonding in a bridge‐like fashion on the Cu_6_ copper core. Upon relaxation, we find that the bond lengths agree with the experimentally obtained crystal structure (Figure S7 and Table S3, Supporting Information). On the other hand, the Cu_6_^−2^ cluster has much shorter Cu—Cu bond lengths than its counterpart, which indicates the Cu_6_ core may be fluxional, meaning with the addition or removal of charge there is a slight change in the geometry of the core (Table S3, Supporting Information). The electronic structure and molecular orbitals (i.e., highest occupied molecular orbital (HOMO) and lowest unoccupied molecular orbital (LUMO)) for Cu_6_^−^ nanocluster is given in **Figure** **4**A. Interestingly, though the Cu_6_(SR)_6_^−^ nanocluster has a small HOMO–LUMO gap of 0.19 eV, the gap between the HOMO and LUMO (i.e., spin‐up states) is 0.9 eV; while the gap between the HOMO and LUMO beta states (spin‐down) is 1.3 eV. However, there is high delocalization centered on the metal core for both the HOMO and LUMO states (Figure 4). From a superatom‐complex point of view (for more information please see Supporting Information), these states can be assigned particular symmetries corresponding to atom‐like states: 1S^2^1P^6^1D^10^… Here, both the HOMO and LUMO states have S‐symmetry. This can be rationalized by the number of electrons withdrawn from the cluster core being equal to the number of ligand surrounding the cluster. Therefore, the 1S state singly occupied. However, if an additional electron is added to the cluster, that is forming Cu_6_(SR)_6_^−2^, one should observe superatom shell closing for the S‐state. In fact, investigation of the orbitals and electronic structure of the Cu_6_(SC_7_H_4_NO)_6_^−2^ reveals that the 1S state is fully occupied (HOMO) and the LUMO has P‐character (Figure S8, Supporting Information) and the HOMO–LUMO gap subsequently increases to 1.17 eV, which indicates it may be possible for the Cu_6_(SC_7_H_4_NO)_6_^−2^ to exist in solution.

With the identification of the electronic structure, we turn our attention to the understanding the identified absorption peaks at 222 and 284 nm. Recall that both *q* = −1 and *q* = −2 could exist in solution. However, both exhibit peaks at 284 nm. Interestingly, the largest peak in the spectra at approximately 300 nm originates from a low‐lying state which has density on the metal copper core and ligand to a high state with induced density only on the ligands (Figure S9, Supporting Information). Similarly, the peak observed at 222 nm involves a low‐lying state, with the induced density residing on only the ligand (Figure S10, Supporting Information). We found similar results for the *q* = −1 cluster. The broad peak in the simulated spectra has been identified as weak excitations originating from the HOMO and atomic d‐states to higher ligand states.

Previous studies into the excitation profile for hexanuclear Cu(I) clusters have proposed metal‐to‐ligand charge transfer (MLCT) as being a key component for absorption (and emission). Our analysis here illustrates that there are indeed key ligand states involved in the absorption profile. However, the excitation profile for hexanuclear Cu‐clusters may be more complex than a simplistic MLCT, but could be considered as ligand/core‐to‐ligand charge transfer. Further, by comparison, the excitation profile of thiol‐protected Cu‐nanoclusters are equally complex as those found for larger thiolate‐protected gold and sliver nanoclusters, and ligand‐protected main‐group systems^12^ by involving states where density is located on the metal core, organic ligand, and even hybrid metal–ligand states. Although the broad peak is not seen in this study, it has been observed in previous studies on hexanuclear systems albeit with very low intensity. The weak intensity of the peak along with possible overestimation of theory could explain the absence of the broad peak from the experimental spectra.

### 2.2 Electrochemical Detection of Hydrogen Peroxide with the Cu_6_ Nanoclusters

**Figure 5:**
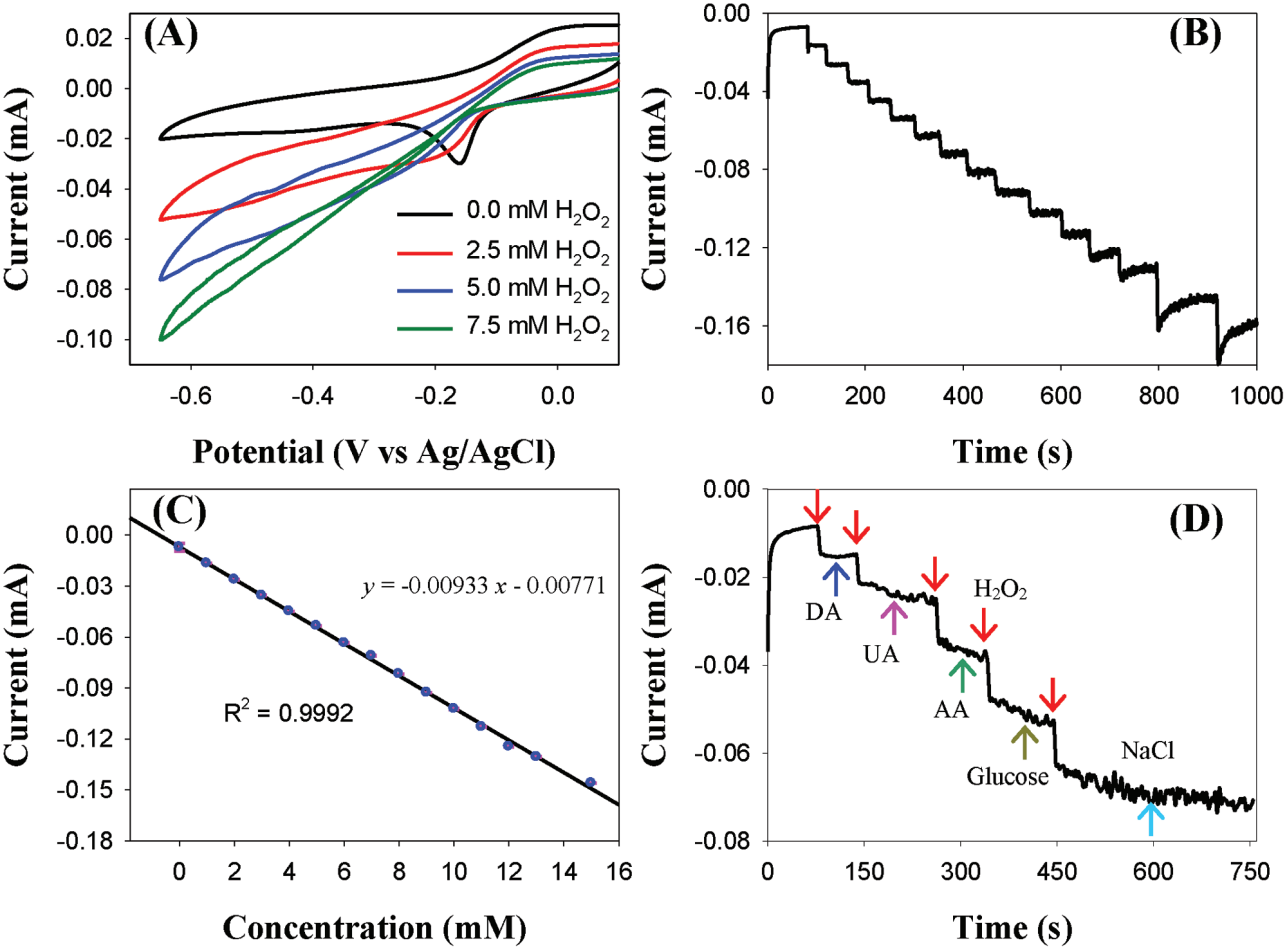
A) CVs of the Cu_6_ clusters in 0.1 m PBS with the absence and presence of H_2_O_2_ at different concentrations of 2.5 × 10^−3^, 5 × 10^−3^, and 7.5 × 10^−3^ m. Scan rate: 100 mV s^−1^. B) The amperometric *i*–*t* curve of the Cu6 clusters upon successive addition of H_2_O_2_ recorded at −0.4 V. C) The linear relationship between responding current and concentration of H_2_O_2_. D) The amperometric responses recorded on alternate addition of interfering chemicals and H_2_O_2_ in 0.1 m PBS. The concentration of the interfering chemicals is 2 × 10^−3^ m, while the concentration of H_2_O_2_ is 1 × 10^−3^ m.

Eventhough the Cu_6_(C_7_H_4_ONS)_6_ cluster can be considered as a ligand‐protected system, the two copper atoms at opposing vertices in the octahedron are exposed, which suggests the cluster may be used in the electrochemical detection of small moleuclues. Therefore, we investigated Cu_6_ clusters for electrochemical detection of H_2_O_2_. **Figure** **5**A shows the CVs of Cu_6_/GC electrode in 0.1 m PBS with different concentrations of H_2_O_2_. It can be seen that obvious reduction current appears after the introduction of H_2_O_2_ and the reduction current increases with the increase of H_2_O_2_ concentration. Such CV features indicate the high electrocatalytic activity of the Cu_6_ clusters for H_2_O_2_ reduction. The amperometric response of Cu_6_ clusters to the successive addition of H_2_O_2_ was investigated at −0.4 V. From the obtained *i*–*t* curve (Figure 5B), it can be clearly seen that the reduction current increases with successive addition of H_2_O_2_ and the current can reach rapidly a steady‐state, indicating the sensitive and fast response of the Cu_6_ clusters to the concentration change of H_2_O_2_. The linear relationship between responding current and concentration of H_2_O_2_ is displayed in Figure 5C. The limit of detection (LOD) was estimated to be 1.8 × 10^−6^ m based on the three times the standard deviation for the average measurement of blank sample (LOD = 3σ s^−1^). Meanwhile, from the fitting curve, a linear range from 1.8 × 10^−6^ to 15 × 10^−6^ m was obtained, which is much wider than some previously reported sensing materials.^13^ It is worthy to note that both of the LOD and linear range are superior to the previously reported Cu_2_O nanomaterials, which may attribute to the tiny size and unique structure of the present copper clusters.^14^ Further investigation into sensing ability of the copper cluster toward hydrogen peroxide was conducted under the influence of other analytes, including glucose, ascorbic acid (AA), dopamine (DA), uric acid (UA), and NaCl. As shown in Figure 5D, almost no change of reduction current can be detected when the interfering chemicals are added, indicating the excellent anti‐interference ability of the copper cluster and the high sensing selectivity of Cu_6_ cluster toward H_2_O_2_. The high detection selectivity could be ascribed to the different reduction potential of H_2_O_2_ electrocatalyzed by the Cu_6_ clusters compared to other studied analytes. The high sensitivity, high selectivity, and wide linear range suggest that the present copper cluster could be a novel sensing material for H_2_O_2_ detection. To investigate the stability of the present Cu_6_ clusters, accelerated durability test (ADT) was conducted in the electrochemical system. As shown in Figure S11 of the Supporting Information, the CV curve shows no much change after 100 cycles, indicating the good electrochemical stability of the Cu_6_ clusters under the present experimental conditions.

## 3 Conclusion

In this paper, we report on the synthesis and characterization of the thiolate‐protected hexanuclear copper nanocluster Cu_6_(C_7_H_4_NO_S_)_6_^−^. The crystal structure of the Cu_6_ clusters with well‐defined metal‐atom packing and metal–ligand bonding have been undoubtedly determined from single crystal analysis. The Cu_6_ nanocluster features a distorted octahedron with thiolates bound through a simple bridge bonding motif. Computational analysis provided elucidation into the open‐shell nature of the cluster and the electronic structure of the hexanuclear cluster. Further analysis revealed that the experimentally observed peaks are comprised of mainly hybridized core/ligand states to ligand states. Moreover, the Cu_6_ clusters also show excellent performance in the detection of H_2_O_2_ with high selectivity and sensitivity, suggesting the potential applications as novel nonenzymatic chemical sensors for H_2_O_2_. These results provide the opportunity for understanding the structures and properties of metal nanoclusters other than Au and Ag. Our findings could lay a solid foundation for the future exploration of non‐noble metal nanoclusters, including synthesis, crystal structure, property, and application.

## 4 Experimental Section

*Chemicals and Materials*: Copper (II) 2,4‐pentanedionate (Cu(acac)_2_, 98%, Alfa Aesar), 2‐mercaptobenzoxazole (C_7_H_5_NOS, MBO, 98%, Alfa Aesar), sodium borohydride (NaBH_4_, 98%, Acros Organic), Acetonitrile (A.R., Urchem), methanol (A.R., Beijing Chemical Works), acetone (A.R., Beijing Chemical Works), acetonitrile‐d_3_ (99.9% atom D, Acros Organics), dichloromethane (A.R., Beijing Chemical Works), and all other chemicals (including NaH_2_PO_4_, Na_2_HPO_4_, 30% H_2_O_2_, ascorbic acid, uric acid, dopamine, NaCl) were purchased from Beijing Chemical Reagent. All chemicals are commercially available and used without further purification. Ultrapure water (18.3 MΩ cm) was used throughout the whole experiments. Furthermore, all the glassware was washed by Aqua Regia (HCl/HNO_3_ with a volume ratio of 3:1) and copiously rinsed with ethanol and ultrapure water.

*Syntheses of Cu_6_(C_7_H_4_NOS)_6_ Nanoclusters*: The synthesis of copper nanoclusters in this study was based the modified procedure for the synthesis of silver clusters.^15^ The whole experimental process was conducted at room temperature. Initially, 0.045 mmol, 11.9 mg of copper (II) 2,4‐pentanedionate was introduced to a flask containing 7.2 mL acetonitrile under constant stirring for 10 min. 7.12 mg of 2‐mercaptobenzoxazole was then mixed with the above solution and the mixture was left for reaction for another 15 min. Meanwhile, 10.8 mg of sodium borohydride was dissolved in acetonitrile within an hour. Subsequently, the above two solutions were mixed in a flask, and was left under stirring for 3 h. The color of the mixed solution changed from light green to yellow, and then deep yellow. After that, acetonitrile solvent was removed by rotary evaporation. The obtained yellow solid was washed twice by diethyl ether in order to eliminate the unreacted thiol and the possible disulfide byproducts. The product was then extracted by dichloromethane. The crystallization of Cu_6_(C_7_H_4_NOS)_6_ clusters was performed in the mixture of acetonitrile and acetone at 4 °C for a month. High‐quality millimeter sized single crystals were obtained and used for the further analysis and characterization.

*Material Characterization*: UV–vis spectra were recorded on a UV‐3000PC Spectrophotometer (Shanghai Manada Instrmenta Co., Ltd.). XPS measurements were performed by using AVG Thermo ESCALAB 250 spectrometer (VG scientific) operated at 120 W. Electrospray ionization mass spectrometry (ESI‐MS) measurements were conducted on a LTQ linear ion trap mass spectrometer (Thermo, San Jose, CA, USA), equipped with a conventional ESI source. Fourier‐transformed infrared spectroscopy (FTIR) study was conducted with a VERTTEX 70 FTIR (KBr wafer technique). ^1^H NMR was carried out on an Avavce III HD 500 (Switzerland, Bruker). X‐ray single crystal diffraction was performed on a Bruker Apex II CCD diffractometer with graphite‐monochromated Mo K*а* radiation (λ = 0.71073 Å). The elements of the product were analyzed by an Inductively Coupled Plasma Mass Spectrometry (ICP‐MS, ThermoScientific Xseries 2). *Zeta*‐potential test was measured on the Malven Nano ZS90.

*Electrochemical Detection of Hydrogen Peroxide*: All measurements were carried out on a CHI 750D electrochemical workstation in a standard three‐electrode cell at room temperature. For the electrochemical detection, a glassy carbon (GC) electrode was first polished with alumina slurries (0.05 μm) and then cleaned by successive sonication in ultrapure water, ethanol and acetone for 10 min. 20 μL of acetonitrile solution of copper cluster (0.5 mg mL^−1^) was then dropcast onto the clean GC surface by a Hamilton microliter syringe. The particle film was dried by a gentle nitrogen stream. The Cu nanoclusters‐modified GC (Cu_6_/GC) was used as working electrode. An Ag/AgCl (in saturated KCl solution) electrode and Pt coil were used as the reference and counter electrodes, respectively. In the present study all electrode potentials are referred to the Ag/AgCl electrode and readout currents were given without any iR drop correction (ohmic drop from solution resistance). The electrolyte solution (0.1 m phosphate buffer solution) was bubbled with ultrahigh purity nitrogen for at least 15 min prior to the electrochemical measurements. Amperometric *i*–*t* curves recorded at 0.4 V were utilized to acquire the linear relationship of responding current and concentration of H_2_O_2_.

*Computational Methods*: Calculations of the Cu_6_(C_7_H_4_NOS)_6_^−^ cluster have been performed with the density functional theory code GPAW,^16^ which implements the projector‐augmented wave method in a real‐space grid. The grid spacing in this work was 0.2 Å, with the cluster surrounded by 6 Å of vacuum in all directions. Cu(3d^10^4s^1^), S(3s^2^3p^4^), N(2s^2^2p^3^), C(2s^2^2p^2^), O(2s^2^2p^4^), H(1s^1^) electrons were regarded as the valence, and the PAW setups for Cu included scalar‐relativistic corrections. Total energies were evaluated at the GGA‐PBE level (gradient‐corrected functional of Perdew, Burke, and Ernzerhof).^17^ Starting from the experimentally obtained crystal structure, all of the atoms were relaxed during the geometry optimization until the maximum force acting on atoms below 0.05 eV Å^−1^ for two different charge states of the Cu_6_‐nanocluster, that is *q* = −1 and −2.

To analyze the ground‐state electronic structure was performed by projecting Kohn–Sham (KS) wave functions onto the atomic orbitals of PAW reference atoms, i.e., by calculating the atomic orbital partial density of states. In addition, the electronic structures were analyzed by applying projection onto “superatomic states,” i.e., by projecting KS wave functions onto spherical harmonics YLM (L ≤ 9) centered at the center of mass of the metal core, and then integrating up to the core radius. For detailed information on this analysis please see Supporting Information.

To analyze the optical spectra we used the Lr‐TDDFT module in GPAW with similar parameters as explained above for *q* = −1. The energy cutoff for KS eigenvalue differences was 5 eV. The optical spectra were folded with a Gaussian with a width of 0.2 eV. In order to analyze the role of the solvent on the optical properties, we performed additional calculations using the conductor‐like screening model (COSMO)^18^ as implemented in Amsterdam Density Functional program (ADF).^19^ The precise details of solvent calculations and functional comparisons are given in the Supporting Information.

## Supporting information

As a service to our authors and readers, this journal provides supporting information supplied by the authors. Such materials are peer reviewed and may be re‐organized for online delivery, but are not copy‐edited or typeset. Technical support issues arising from supporting information (other than missing files) should be addressed to the authors.

Supplementary
