# Supplementary material for: Single Crystal Sub‐Nanometer Sized Cu6(SR)6 Clusters: Structure, Photophysical Properties, and Electrochemical Sensing
Source: Adv Sci (Weinh). 2016 Jul 14;3(12):1600126. doi: 10.1002/advs.201600126 (PMC5157172; doi:10.1002/advs.201600126)
Supplement: Supplementary file 1 — Supplementary [file ADVS-3-0-s001.pdf]

Copyright WILEY-VCH Verlag GmbH & Co. KGaA, 69469 Weinheim, Germany, 2016.

ADVANCED SCIENCE
Open Access

Supporting Information

for *Adv. Sci.,* DOI: 10.1002/advs.201600126

Single Crystal Sub-Nanometer Sized $Cu_6(SR)_6$ Clusters: Structure, Photophysical Properties, and Electrochemical Sensing

Xiaohui Gao, Shuijian He, Chunmei Zhang, Cheng Du, Xi Chen, Wei Xing, Shengli Chen, Andre Clayborne, and Wei Chen**

Copyright WILEY-VCH Verlag GmbH & Co. KGaA, 69469 Weinheim, Germany, 2013.

Supporting Information

Single Crystal Sub-Nanometer Sized $Cu_6(SR)_6$ Clusters: Structure, Photophysical Properties, and Electrochemical Sensing

Xiaohui Gao, Shijian He, Chunmei Zhang, Cheng Du, Xi Chen, Andre Clayborne and Wei Chen**

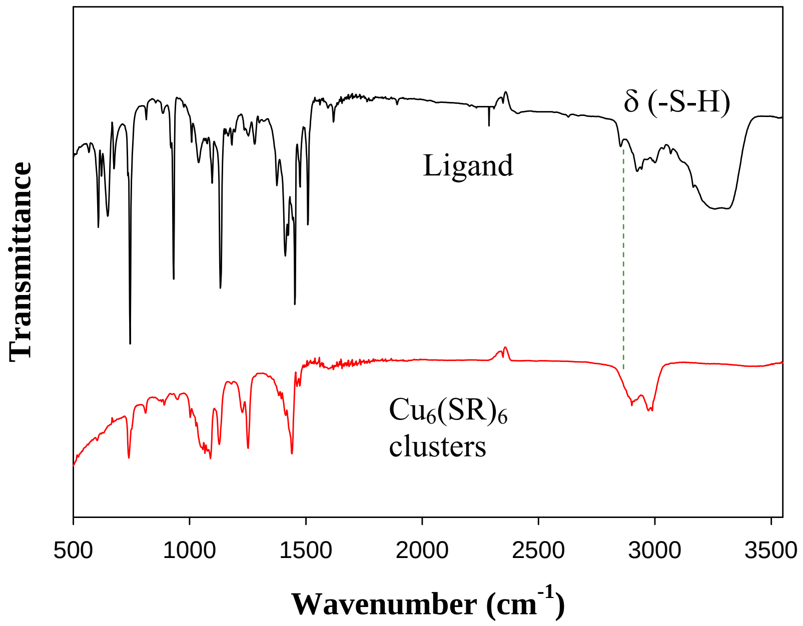

Figure S1 FTIR spectra of $Cu_6(SR)_6$ clusters (red curve) and 2-mercaptobenzoxazole monomer (black curve).

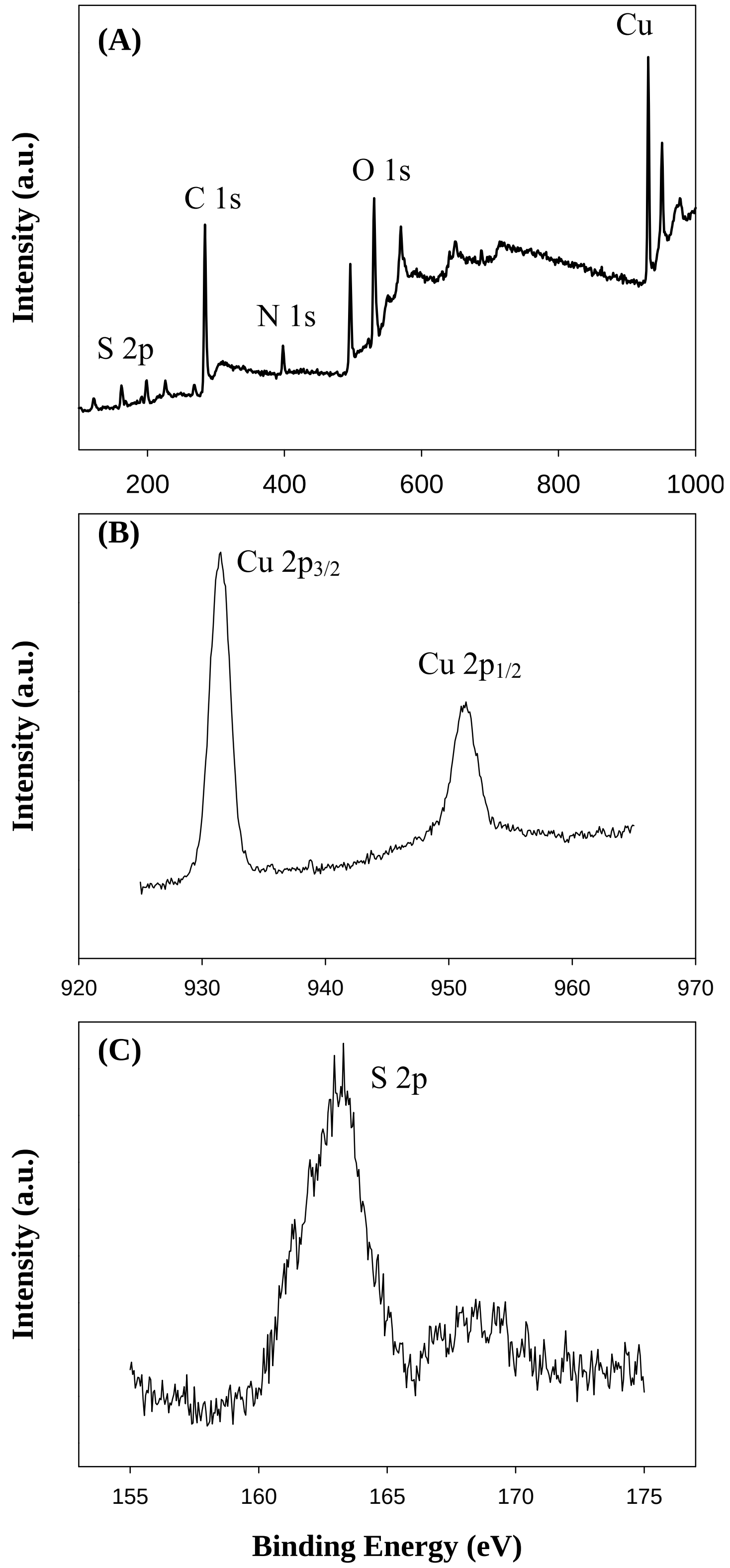

Figure S2 (A) The XPS survey spectrum of Cu_6 clusters. High resolution XPS spectra of Cu 2p (B) and S 2p (C) in the Cu_6 clusters.

(A)

(B)

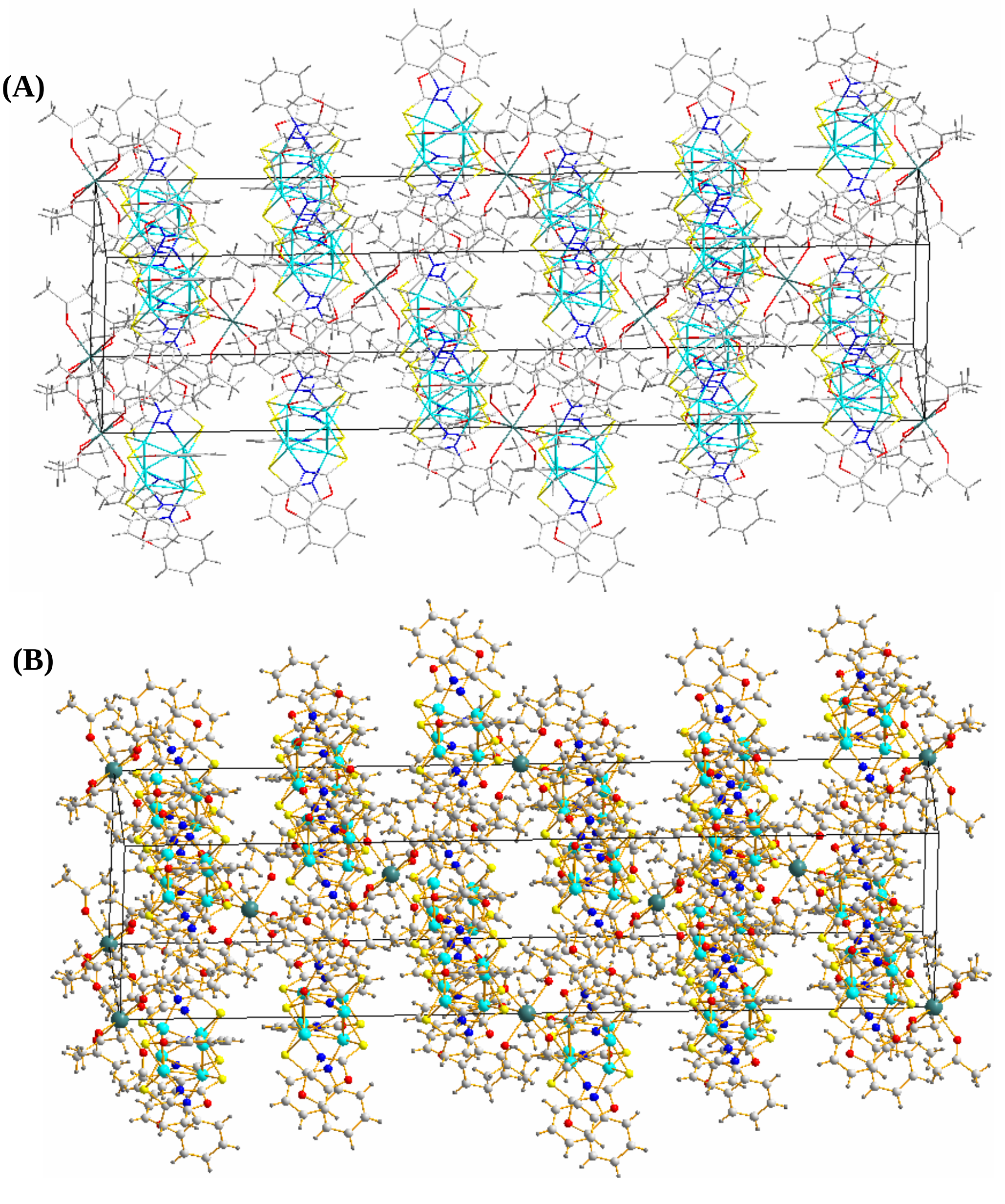

Figure S3 Structure of $Cu_6(SR)_6^-$ clusters with $Na(C_3H_6O)_6^+$ as counterions, showing in stick model (A) and ball-and-stick model (B).

Table S1 Crystal structure data and structure refinement for the $Cu_6(SR)_6$ cluster

Identification code	$Cu_6(SR)_6$
Empirical formula	$C_{60}H_{60}Cu_6N_6NaO_{12}S_6$
Formula weight	1653.73
Temperature	293(2) K
Wavelength	0.71073 A
Crystal system, space group	Hexagonal, R-3c
Unit cell dimensions	a = 15.4894(9) A α= 90 deg.
	b = 15.4894(9) A β= 90 deg.
	c = 49.292(3) A γ = 120 deg.
Volume	10241.8(10) A^3
Z, Calculated density	6, 1.609 mg/m^3
Absorption coefficient	2.087 mm^{-1}
F(000)	5034
Crystal size	0.23 x 0.22 x 0.21 mm
Theta range for data collection	1.73 to 26.00 deg.
Limiting indices	$-19\leq h\leq 18$, $-19\leq k\leq 15$, $-43\leq l\leq 60$
Reflections collected / unique	20322 / 2247 [R(int) = 0.0421]
Completeness to theta = 26.00	100.0 %
Absorption correction	Multi_scan
Max. and min. transmission	0.645 and 0.625
Refinement method	Full-matrix least-squares on F^2
Data / restraints / parameters	2247 / 0 / 140
Goodness-of-fit on F^2	1.039
Final R indices [I>2sigma(I)]	R1 = 0.0305, *w*R2 = 0.0788
R indices (all data)	R1 = 0.0507, *w*R2 = 0.0915
Largest diff. peak and hole	0.493 and -0.289 e.A^-3

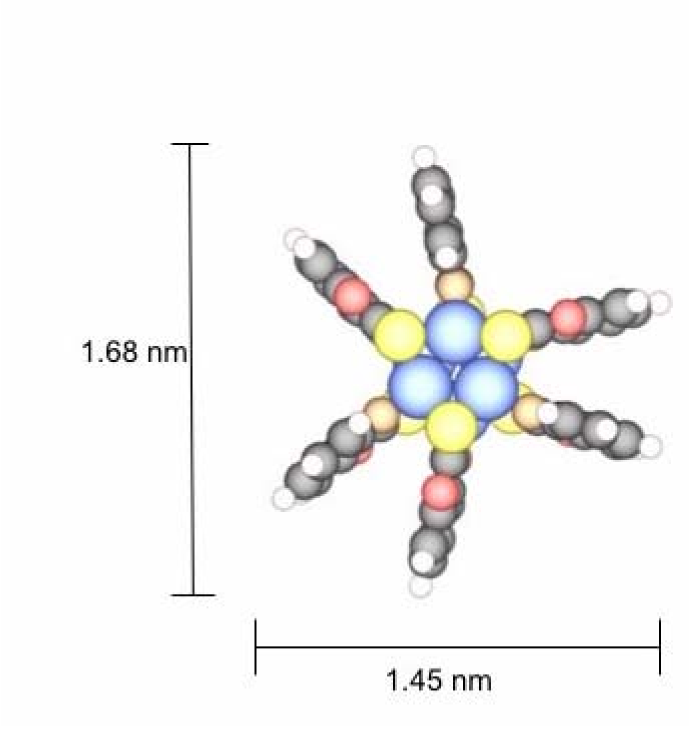

Figure S4 The size of the Cu_6 cluster calculated from theoretical analysis.

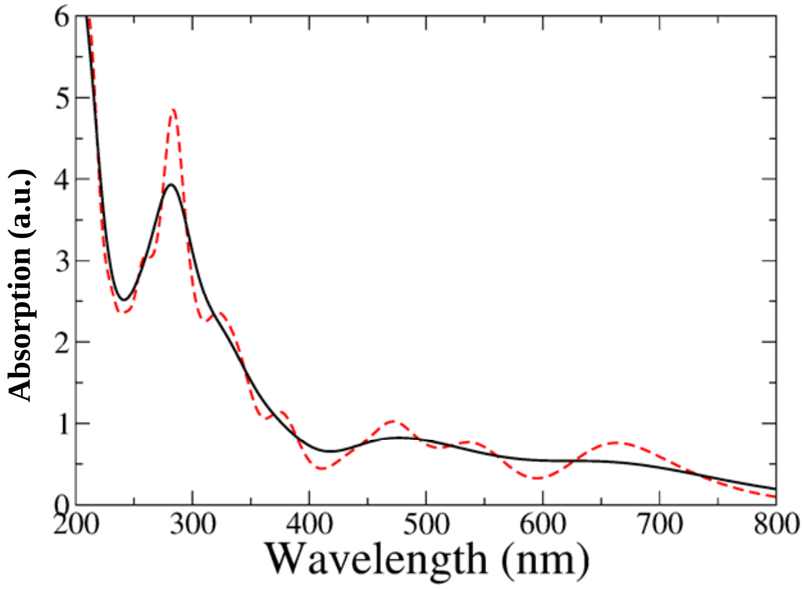

Figure S5 Optical spectra (from 200 – 800 nm) of $Cu_6(SC_7H_4NO)_6{}^{-2}$. The red line is the spectra with an applied gaussian width of 0.1. The black represents the spectra folded with 0.2 gaussian width.

Computational Methods for Optical Analysis in Solution

The calculations for solvent effects on the excitation spectra were performed with the Amsterdam Density Functional (ADF) program[1-3]. A triple zeta basis set of Slater orbitals was employed for H, C, Cu, S, and N in the calculations. Possible effects on the optical spectra by the dicloromethane solution were treated implicitly using the conductor-like screening model (COSMO)[4] as implemented in ADF. The model potential includes the exchange functional of Van Leeuwen and Baerends (LB94)[5]. The calculated optical spectra in the gas-phase and solution can be found in Figure S9. The spectra shape remains the same, however the spectra in solution shift by approximately 0.1 eV.

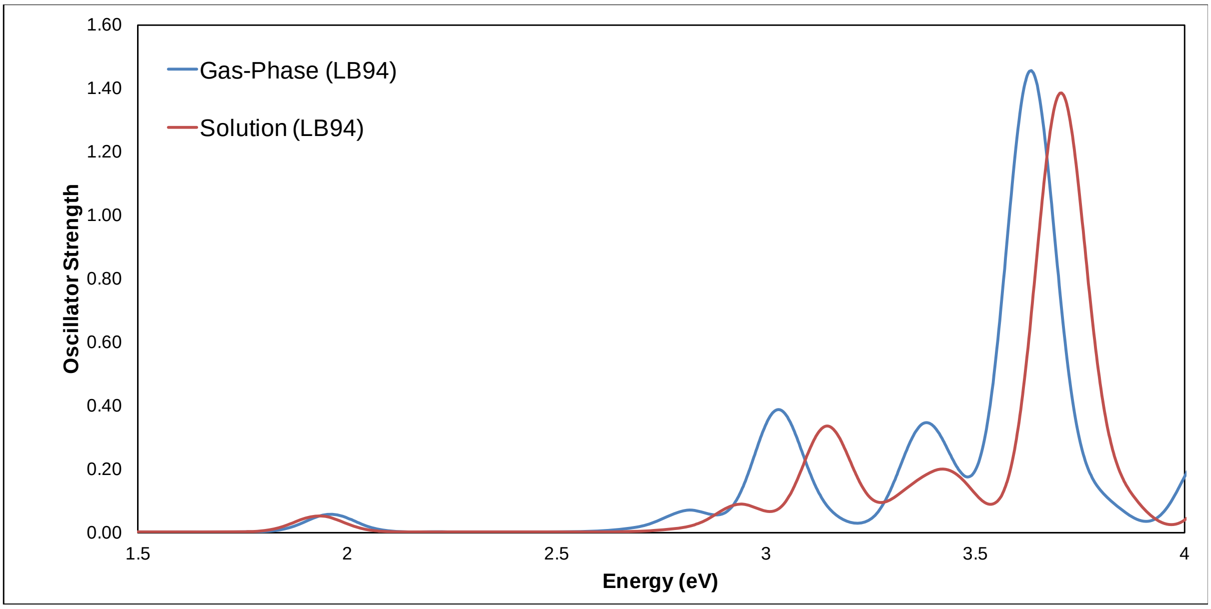

Figure S6 Comparison of the optical spectra in the gas-phase and solution for $Cu_6(SC_7H_4NO)_6^{-1}$.

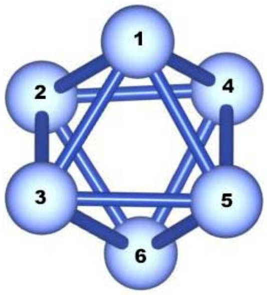

Figure S7 Cu_6 copper core with numbered Cu-atoms for determining the bond lengths found in Table S2 and Table S3.

Table S2 Experimental Bond length data of the $Cu_6(SR)_6$ cluster

Atoms	Length (Å)
**Cu(1)–Cu(2), Cu(1)–Cu(4), Cu(2)–Cu(4), Cu(3)–Cu(5), Cu(3)–Cu(6), Cu(5)–Cu(6),**	2.581
**Cu(1)–Cu(3), Cu(2)–Cu(6) Cu(4)–Cu(5)**	2.776
**Cu(1)–Cu(5), Cu(2)–Cu(3) Cu(4)–Cu(6)**	2.883
**Cu-S**	2.33

Superatom Complex Model and Theoretical Bond analysis

In the following section, we would like to briefly explain the superatom complex model[3] to aid the reader in understanding this model. The superatom complex model originates from the jellium model for metal clusters. The jellium model incorporates a positively charged (spherical or non- spherical) background potential to solve the Schrödinger equation, which results in discrete energy levels of the delocalized ("metallic") electrons that correspond to angular momentum shells (in the spherical case the shells are labeled as: $1S^2$ $1P^6$ $1D^{10}$ $2S^2$ $1F^{14}$, $2P^6$, $1G^{18}$...). In cases where the electronic shells are filled, the number of electrons (n_e) corresponds to one of the "magic" numbers (n_e = 2, 8, 18, 20, 40, 58...), and a large gap between the highest occupied molecular orbital and lowest unoccupied molecular orbital (HOMO-LUMO gap) appears in the electronic shell structure. The superatom complex model uses the jellium model as a foundation, but takes into account the electron withdrawing effect of the organic or organometallic ligand. For example, if one has a monolayer-protected cluster with the formula $[A_NL_X]^z$, and each ligand withdraws electrons from the cluster core, an equation for the number of delocalized electrons can be determined via,

$$n_e = N_A v_A - X_L w_L - z \qquad (1)$$

where N_A is the number of atoms, v_A is the number of valence electrons by the atom, X_L is the number of ligands, w_L is the number of electrons withdrawn by the ligand and z is the overall charge of the cluster. For the $Cu_6(SC_7H_4NO)_6^{-1}$, $n_e = 1$, while for $Cu_6(SC_7H_4NO)_6^{-2}$ $n_e = 2$; which results in the electronic configurations of $1S^1$ and $1S^2$ respectively.

The hollow nature of the Cu_6 core (Figure S4) can allow for some fluxinal behavior. The fluxinal behavior can be illustrated within small changes within the Cu-Cu bond distances within the copper core due to the different charge states, that is q = -1, -2, etc. This effect is known for various hollow-cage systems such as Zintl clusters[4] and metalloid nanoclusters,[5] which can be stable with different charge states. Table S3 gives the theoretical bond distances for the relaxed structures for both the anion and doubly negatively charged system.

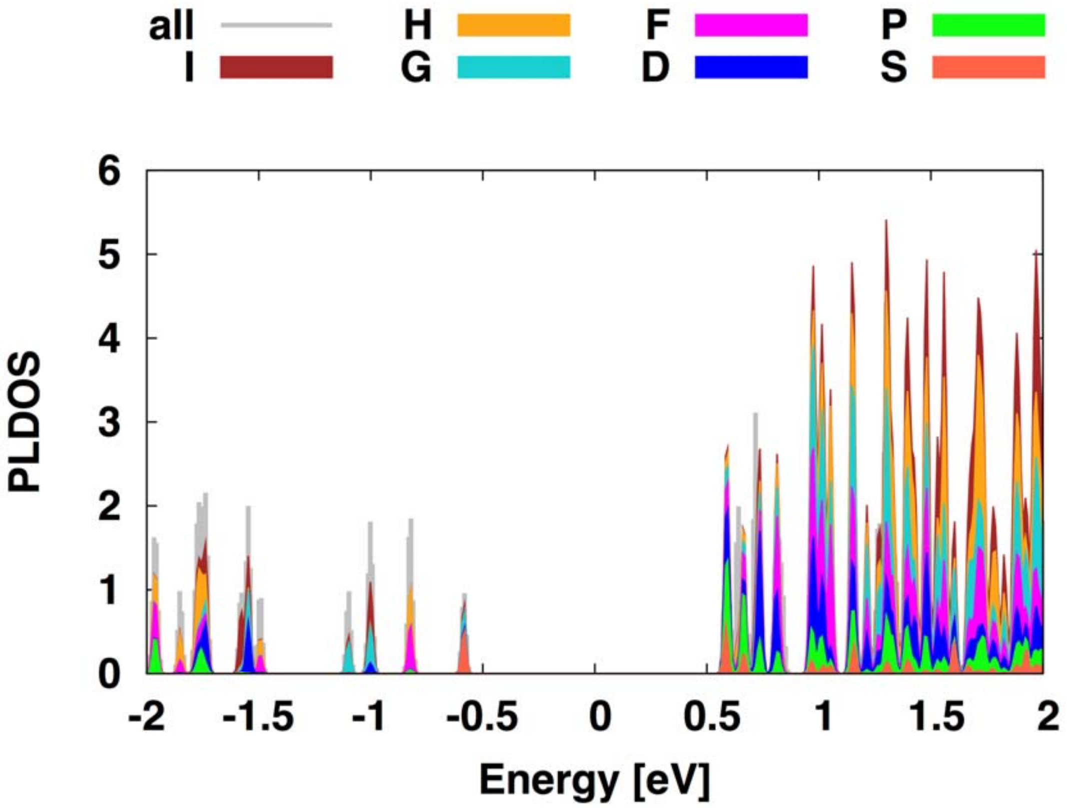

Figure S8 Projection of the Kohn-Sham electron states to spherical harmonics in the $Cu_6(SC_7H_4NO)_6{}^{-2}$.

Table S3 Theoretical Bond lengths (in Å) of the Cu_6 core for $Cu_6(SC_7H_4NO)_6{}^{-q}$.

**Atoms**	**q = -1**		**q = -2**
	**Theory**	**Experiment**	
**Cu(1) – Cu(2)**	2.53	2.58	2.60
**Cu(1) – Cu(3)**	2.68	2.77	2.50
**Cu(1) – Cu(4)**	2.53	2.58	2.53
**Cu(1) – Cu(5)**	2.74	2.88	2.48
**Cu(1) – Cu(6)**	3.71	3.82	3.55
**Cu(2) – Cu(3)**	2.75	2.88	2.52
**Cu(2) – Cu(4)**	2.57	2.58	2.49
**Cu(2) – Cu(6)**	2.65	2.77	2.48
**Cu(3) – Cu(5)**	2.57	2.58	2.50
**Cu(3) – Cu(6)**	2.57	2.58	2.59
**Cu(4) – Cu(5)**	2.67	2.77	2.61
**Cu(4) – Cu(6)**	2.80	2.88	2.49
**Cu(5) – Cu(6)**	2.56	2.58	2.52

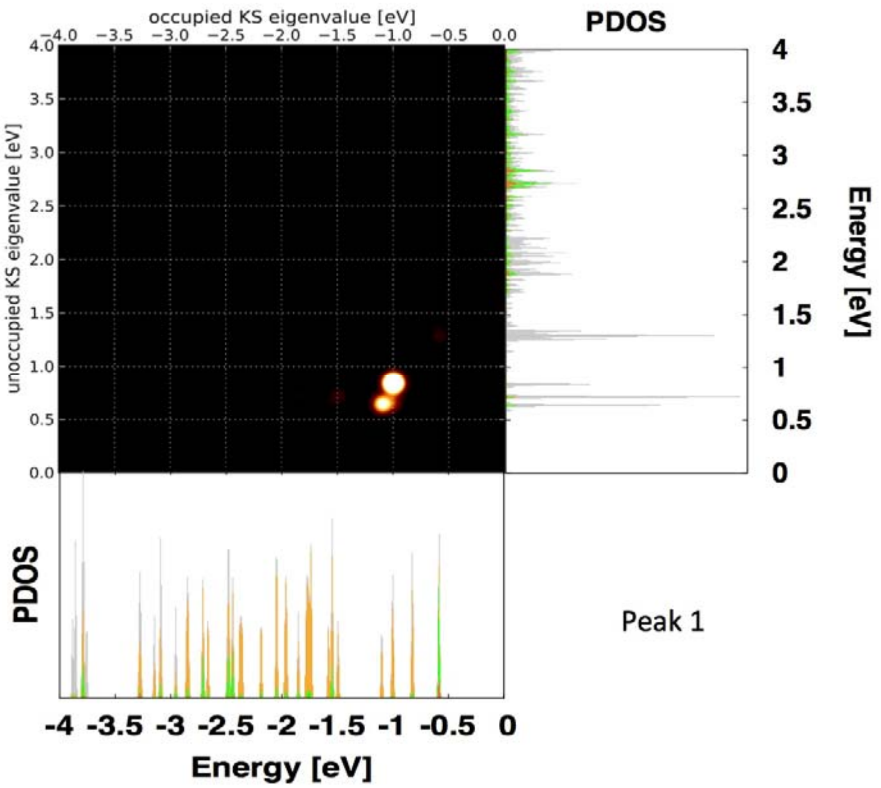

Figure S9 Transition contribution map for excitation at 663 nm. The density of states (PDOS) gives the total density (grey), Cu s, p, and d state in red, green, and orange respectively.

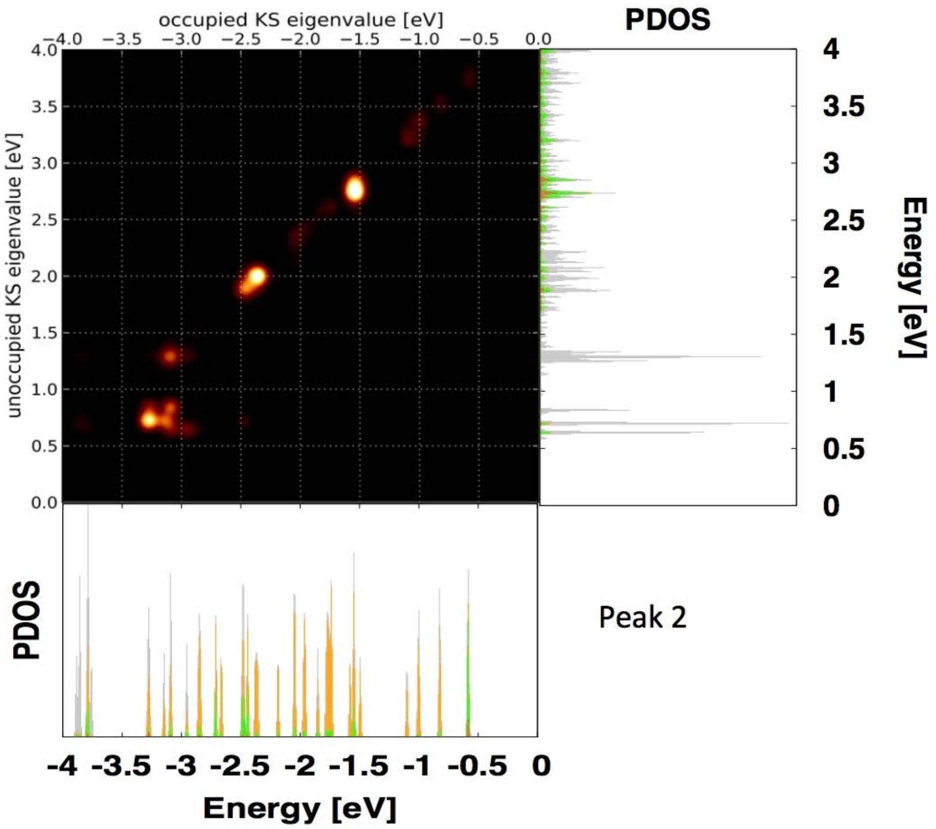

Figure S10 Transition contribution map of initial peak at 284 nm. For more information, please refer to Figure S8.

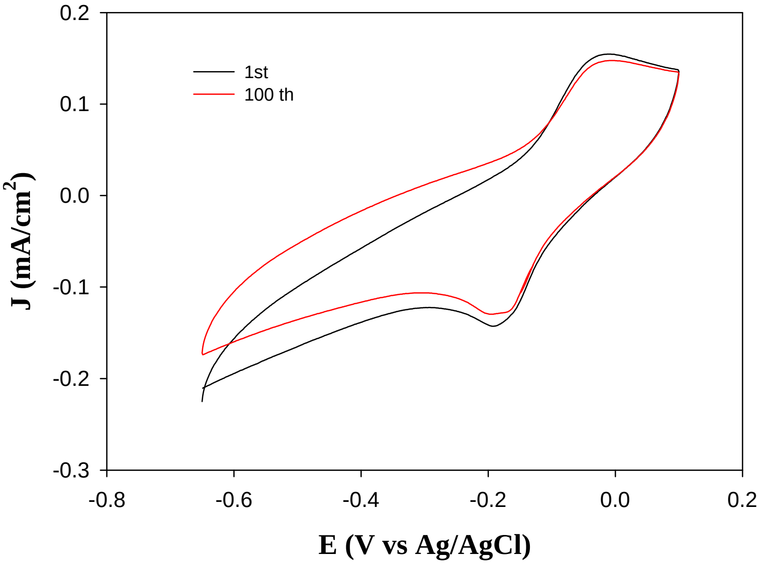

Figure S11 Cyclic voltammetry curves of the Cu_6 clusters in 0.1 M PBS for an accelerated durability test.

References for Supporting Information

(1) Pye, C. C.; Ziegler, T. *Theor. Chem. Acc.* **1999,** *101*, 396.

(2) ADF2014, SCM, Theoretical Chemistry, Vrije Universiteit, Amsterdam, The Netherlands, http://www.scm.com.

(3) Walter, M.; Akola, J.; Lopez-Acevedo, O.; Jadzinsky, P. D.; Calero, G.; Ackerson, C. J.; Whetten, R. L.; Gronbeck, H.; Hakkinen, H. *P. Natl. Acad. Sci. USA* **2008,** *105*, 9157.

(4) Clayborne, P. A.; Hakkinen, H. *Phys. Chem. Chem. Phys.* **2012,** *14*, 9311.

(5) Schnepf, A. *Coordin. Chem. Rev.* **2006,** *250*, 2758.
